# Epithelium-Free Area in The Thymic Cortex of Rats

**DOI:** 10.1155/1993/90759

**Published:** 1993

**Authors:** Joost P. Bruijntjes, C. Frieke Kuper, Joke E. Robinson, Henk-Jan Schuurman

**Affiliations:** 1 TNO Toxicology and Nutrition Institute Zeist The Netherlands; 2 National Institute of Public Health and Environmental Protection Bilthoven The Netherlands; 3 Departments of Internal Medicine and Pathology University Hospital Utrecht Utrecht The Netherlands; 4 National Institute of Public Health and Environmental Protection P.O. Box 1 Bilthoven 3720 BA The Netherlands

**Keywords:** Epithelium-free compartment, thymus cortex, thymus subcapsule, histochemistry, rat, lymphocyte differentiation

## Abstract

The histology of epithelium-free areas in the subcapsular region of the thymus was studied in Wistar rats. Lymphocytes in these areas were CD4/CD8 double-positive, TCR α/β positive in low intensity, and in CD5 labeling either negative or positive in low intensity. There was a high proliferative activity as assessed by bromodeoxyuridine incorporation *in vivo* and detected by immunohistochemistry. Various macrophage types were observed. They were either large and round to slightly dendritic, or small and dendritic. Most large cells were positive for MHC Class II, and labeled by the antimacrophage antibodies ED1 and ED2. A few cells were strongly positive for Sudan black, Oil red O, nonspecific esterase, and acid phosphatase; they resembled the large rounded macrophages in the corticomedullary zone, although their MHC Class II and ED2 staining was more intense. A few cells showed features of tingible body macrophages, as they contained cellular debris.

Serial sections showed that epithelium-free areas run from the subcapsular area to deep in the cortex, and often border the medulla. This opens the opportunity for immature lymphocytes to move into the medulla and corticomedullary zone without contacting and potential selection with cortical stromal elements other than macrophages in the epithelium-free areas. In this case, the epithelium-free areas may offer a separate intrathymic pathway for T lymphocytes.


*Developmental Immunology*, 1993, Vol. 3, pp. 113–122
Reprints available directly from the publisher
Photocopying permitted by license only

© 1993 Harwood Academic Publishers GmbH
Printed in the United States of America

### Epithelium-Free Area in the Thymic Cortex of Rats

JOOST P. BRUIJNTJES,† C. FRIEKE KUPER,*‡ JOKE E. ROBINSON,‡ and HENK-JAN SCHUURMAN‡§

*†TNO Toxicology and Nutrition Institute, Zeist, The Netherlands.*
*‡National Institute of Public Health and Environmental Protection, Bilthoven, The Netherlands.*
*§Departments of Internal Medicine and Pathology, University Hospital Utrecht, Utrecht, The Netherlands.*

The histology of epithelium-free areas in the subcapsular region of the thymus was studied in Wistar rats. Lymphocytes in these areas were CD4/CD8 double-positive, TCR $\alpha/\beta$ positive in low intensity, and in CD5 labeling either negative or positive in low intensity. There was a high proliferative activity as assessed by bromodeoxyuridine incorporation *in vivo* and detected by immunohistochemistry. Various macrophage types were observed. They were either large and round to slightly dendritic, or small and dendritic. Most large cells were positive for MHC Class II, and labeled by the antimacrophage antibodies ED1 and ED2. A few cells were strongly positive for Sudan black, Oil red O, nonspecific esterase, and acid phosphatase; they resembled the large rounded macrophages in the corticomedullary zone, although their MHC Class II and ED2 staining was more intense. A few cells showed features of tingible body macrophages, as they contained cellular debris.

Serial sections showed that epithelium-free areas run from the subcapsular area to deep in the cortex, and often border the medulla. This opens the opportunity for immature lymphocytes to move into the medulla and corticomedullary zone without contacting and potential selection with cortical stromal elements other than macrophages in the epithelium-free areas. In this case, the epithelium-free areas may offer a separate intrathymic pathway for T lymphocytes.

KEYWORDS: Epithelium-free compartment, thymus cortex, thymus subcapsule, histochemistry, rat, lymphocyte differentiation.

### INTRODUCTION

The thymus harbors various compartments or microenvironments, based on lymphoid- and nonlymphoid-cell characteristics. Among these are areas devoid of stromal elements. Adjacent to the capsule and septa of the thymus, areas can be discerned where there are no epithelial cells. These so-called epithelium-free areas (EFA) show an abundance of lymphocytes (rat: Duijvestijn et al. 1982; mouse: Van Ewijk, 1984; Godfrey et al., 1990; man: epithelium-free areas in the inner cortex; Von Gaudecker, 1986). The occurrence and extent of these EFA varies between strains of rats. In the thymus of WAG/Rij rats, such areas have not been observed, whereas in diabetes-prone (DP) and diabetes-resistant (DR) BB rats, they make up 5% and 3% of the thymic volume, respectively (Rozing et al., 1989). The thymus of BB rats also showed EFA in the medulla and in the corticomedullary region (CMR). Medullary EFA were also found immediately after and during recovery from Cyclosporin treatment (Schuurman et al., 1990). It is questionable whether the areas in the cortex of untreated, healthy rats represent a similar histologic entity as EFA in the medulla and CMR of BB rats and rats after Cyclosporin treatment. This aside, EFA is evidently different from the perivascular space (PVS). PVS are connective tissue regions, containing collagen and matrix, and are as such considered as an extrathymic area (Christensen, 1952; Kendall, 1989). They are lined by fenestrated sheaths of type-1 epithelial cells on a basal lamina.

The occurrence of EFA is dependent on age. EFA in young adult Wistar rats can be fairly extensive, and in rats over 17 months of age, such

*Corresponding author. Present address: National Institute of Public Health and Environmental Protection, P.O. Box 1, 3720 BA Bilthoven, The Netherlands.

areas have not been found (Kuper et al., in press). Instead, perivascular spaces, especially in the CMR, were more prominent. A (transient) increase in PVS volume has also been demonstrated in the human thymus (Steinman, 1986).

The characteristics of EFA were not clearly defined, and their function, if there is any, is unknown. They may be reservoirs for lymphocytes (Van Ewijk, 1984) or proliferation sites of lymphocytes (Duijvestijn et al., 1982; Godfrey et al., 1990). We therefore performed an enzyme- and immunohistochemical study in rats to investigate the lymphoid and nonlymphoid elements in the EFA of the thymus cortex, in order to elucidate possible functions of the compartment. To investigate the proliferative activity in the areas, the thymidine analog bromodeoxyuridine (BrdU) was injected in rats, and the presence of BrdU in thymus was detected immunohistochemically.

TABLE 1
Mouse monoclonal and rabbit polyclonal antibodies used in this study

| Monoclonal | Specificity for thymus cells | Source[a] | Refs[b] |
|---|---|---|---|
| W3/25 | CD4, subset thymocytes | Serotec | 1 |
| MRC OX19 | CD5, subset thymocytes | Serotec | 2 |
| MRC OX8 | CD8, subset thymocytes | Serotec | 1 |
| R73 | T-cell receptor $\alpha/\beta$ chain subset thymocytes | | 3 |
| HIS44 | Most cortical lymphocytes, some medullary lymphocytes | | 4 |
| MRC OX4 | MHC Class II, cortical and medullary epithelium, IDCs, subset macrophages | Serotec | 5 |
| ED1 | Majority of macrophages, IDCs and monocytes | | 6 |
| ED2 | Subset cortical macrophages | | 6 |
| RCK 105 | Keratin 7 (54KD), cortical epithelial cells, and some medullary epithelial cells | | 7, 8, 9 |
| RGE 53 | Keratin 18 (45KD), cortical epithelial cells, and some medullary epithelial cells | | 7, 8, 9 |
| HIS39 | Subcapsular and medullary epithelium | | 10 |
| anti-BrdU | Thymidine analog bromodeoxyuridine | Dakopatts | 11 |
| HIS14 | All B lymphocytes | | 12 |
| **Polyclonal** | **Directed against** | **Source** | |
| Anti-Laminin | Laminin | Dakopatts | |

[a]Commerical sources; for noncommercial sources, see Acknowledgments.
[b]References: 1: Williams et al., 1977; 2: Dallman et al., 1984; 3: Hunig et al., 1989; 4: Kampinga, 1990; 5: McMaster and Williams, 1979; 6: Dijkstra et al., 1985; 7: Moll et al., 1982; 8: Ramaekers et al., 1983; 9: Ramaekers et al., 1987; 10: Kampinga et al., 1987; 11: Gratzner, 1982; 12: Kroese et al., 1987.

### RESULTS

### General Histology

Along the capsule and septa, areas were found that, in H&E-stained sections, were prominent due to their high number of lymphocytes but in which no epithelial cells are evident. The areas were negative for keratin (antibodies: see Table 1; Fig. 1). MHC Class II staining was also negative except for single cells (Figs. 2, and 8; see also what follows under "Macrophages"). Almost no laminin was found, either within the areas or between the areas and the epithelium-containing thymic tissue (Fig. 3). Vascularization was virtually absent. The subcapsular epithelial layer was found between these cortical epithelium-free areas (EFA) and the connective tissue of capsule and septa (Fig. 4). Some of the EFA ran from the capsule to the medulla. Serial sections from one thymus often showed medullary buds bordering the EFA (Fig. 2). The medullary epithelial network extended with cell processes into the areas.

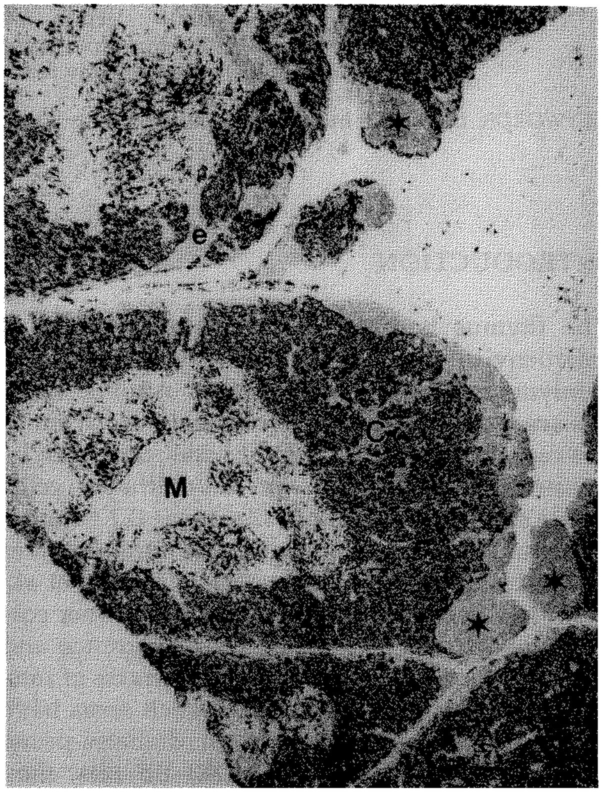

FIGURE 1. A low-power view of the thymus, immunostained for keratin (RGE 53). Several EFA are transected (*), mostly restricted to the outer cortex. One EFA transection (e) extends to the medulla. M: medulla; C: cortex. (×16).

The bordering cortical epithelium was mostly rounded off, with occasionally some dendritic processes extending into the EFA (Figs. 1, 2, and 7). In H&E-stained sections, the flattened cortical epithelium gave the impression of demarcating the rest of the cortex from the EFA. An effect of sex on the presence and size of EFA was not apparent, although it has not been examined systematically.

### Lymphocytes in EFA

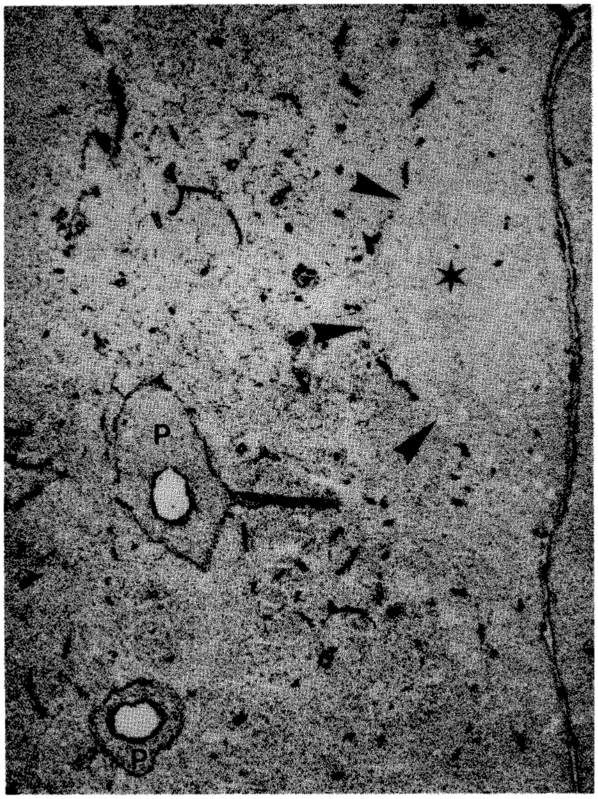

FIGURE 3. Immunostaining for laminin. EFA (*) is not bordered by a laminin layer (arrowheads indicate EFA border). Perivascular spaces (PVS or P) with a laminin layer between the PBS (P) and thymic epithelium are also shown here. (×60).

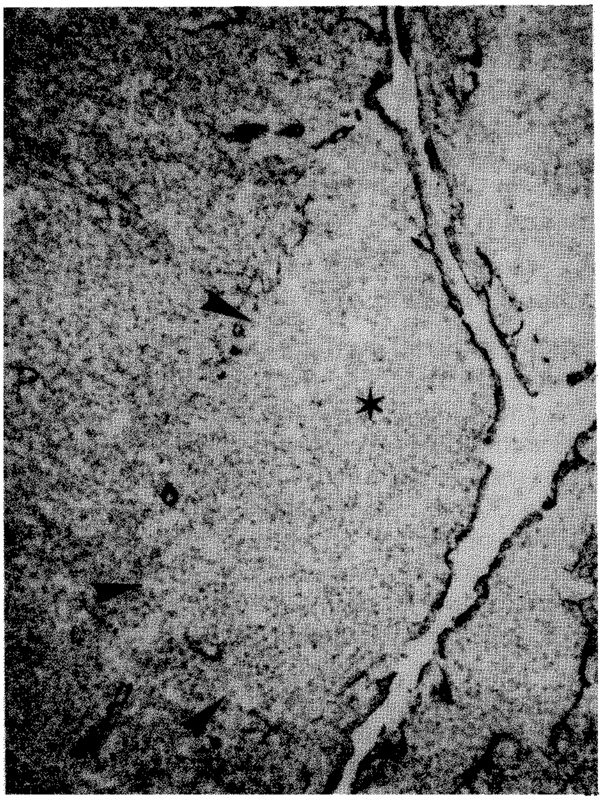

FIGURE 4. Immunostaining for HIS39. The subcapsular epithelium borders EFA (*) (arrowheads indicate EFA border). (×160).

cells with a rounded to dendritic morphology and small dendritic macrophage like cells (Figs. 2 and 8). The large cells showed a confluent Class-II reactivity, comparable to that of medullary IDCs and of single cells in the cortex. ED1 staining (panmacrophage marker, Table 1) showed predominantly large cells with a rounded to slightly dendritic morphology and a few small dendritic cells. There were some large cells with a rounded morphology, and a few small dendritic cells that were ED1-positive but negative or faintly immunoreactive for MHC Class II (Fig. 8). Most ED2-positive cells were large cells with a rounded to dendritic morphology (ED2, cortical macrophage marker, Table 1). In number and cell contour, these cells were comparable to cells identified in ED1 staining. ED2 staining of the large cells was more intense than in the rest of the cortex. In two-color immunohistochemistry for ED1 and ED2, only a few large and small cells were ED1 positive/ED2 negative (Fig. 9).

A few large, rounded cells were strongly positive for Oil red O, Sudan Black, nonspecific esterase (NSE; Fig. 10) and acid phosphatase (AP). Other macrophages were weakly positive for NSE and AP.

### DISCUSSION

Epithelial-free areas (EFA) are found in the outer cortex of the thymus, mainly immediately bordering the subcapsular epithelial-cell layer. They can run deep into the cortex and even reach the medulla. At other sites, medullary buds contact the EFA. Immediate contact between lymphocytes in EFA and cortical and medullary epithelium is feasible, because no basal lamina and connective tissue are found between the epithelium and the EFA. Moreover, in keratin and MHC Class-II labeling, the medullary epithelial lining and, at some places, also the cortical epi-

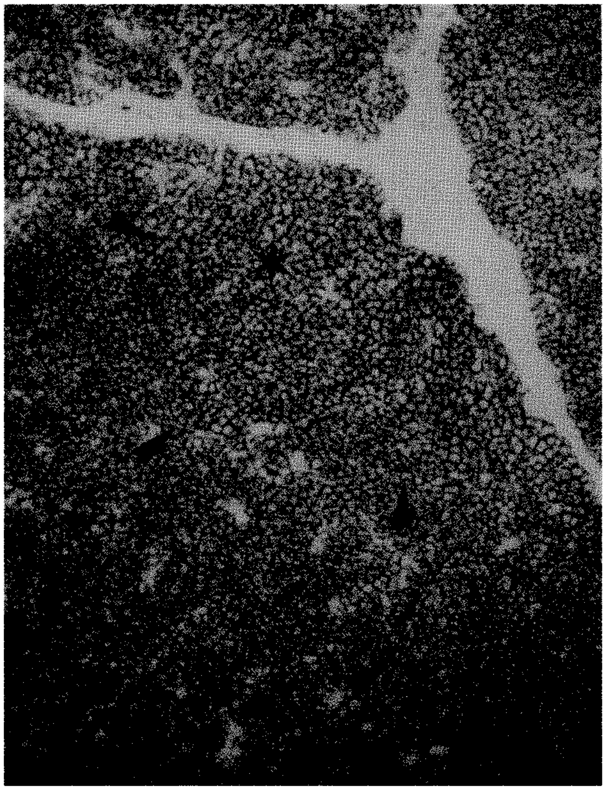

FIGURE 5. Immunostaining for CD8 (OX8). CD8 antibody labeled almost all cells in EFA (*; arrowheads indicate EFA border). (×160).

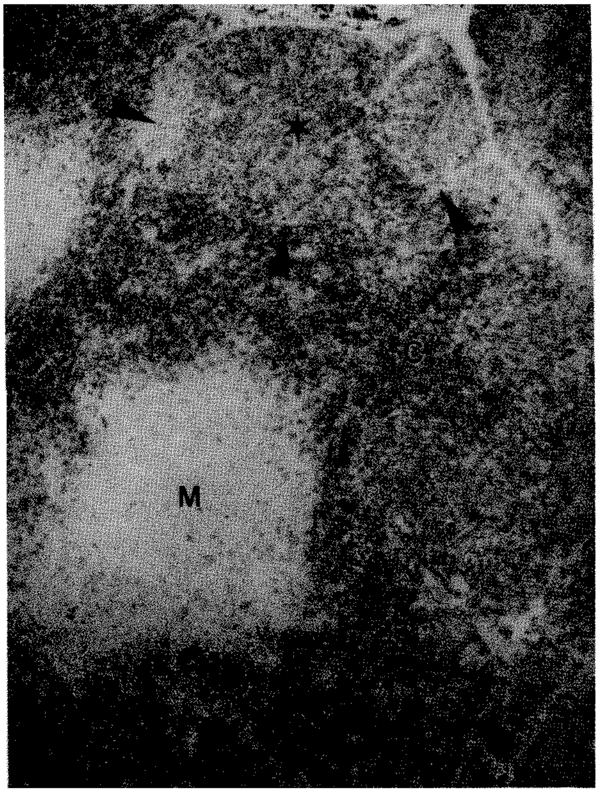

FIGURE 6. Immunostaining for HIS44. Lymphocytes in EFA (*; arrowheads indicate EFA border) are variably stained. M: medulla; C: cortex. (×160).

thelial lining with the EFA suggest that there is free cell movement of lymphocytes between the EFA and the thymic epithelial network. In addition, free cell movement between EFA and CMR appears possible (Figs. 2 and 7).

Serial staining with CD4 and CD8 suggests that the predominant lymphocyte is CD4/CD8 double-positive. This is in accordance with findings of Godfrey et al. (1990) and Rozing et al. (1989). EFA also contain TCR $\alpha/\beta$ positive cells. This implies that lymphocytes in the EFA already passed the first intrathymic development, including TCR gene rearrangement. Godfrey et al. (1990) have suggested that the EFA are isolated "bags" of CD4+/CD8+, proliferating lymphocytes before they contact the thymic stroma. Proliferation in EFA is evident from BrdU labeling, performed in double staining with keratin or MHC Class II and BrdU. Moreover, EFA contain several lymphocytes that are either strongly positive or faintly positive for HIS44. Kampinga (1990) has suggested that lymphocytes lose this marker during intrathymic proliferation. Following his suggestion, the presence of this marker indicates that lymphocytes in the EFA stay a while before proliferation, or stay sufficiently long after proliferation to regain the marker. Boyd and Hugo (1991) have hypothesized that cells in EFA are accumulations of double-positive lymphocytes, which are not under the influence of positive selection, and subsequently die by apoptosis.

In EFA, macrophages with features of TBM are not frequent. However, under "stressfull" conditions, for example, after dexamethason administration, TBM may accumulate in EFA (Fig. 11; unpublished results). Moreover, the large rounded macrophages in EFA, which are strongly positive for Oil red O, NSE, AP and Sudan Black and resemble CMR macrophages (Milicevic et al., 1987; Milicevic and Milicevic, 1989), might be precursors of tingible body macrophages (TBM), because they sometimes contain nuclear debris. Aggregates of the CMR macro-

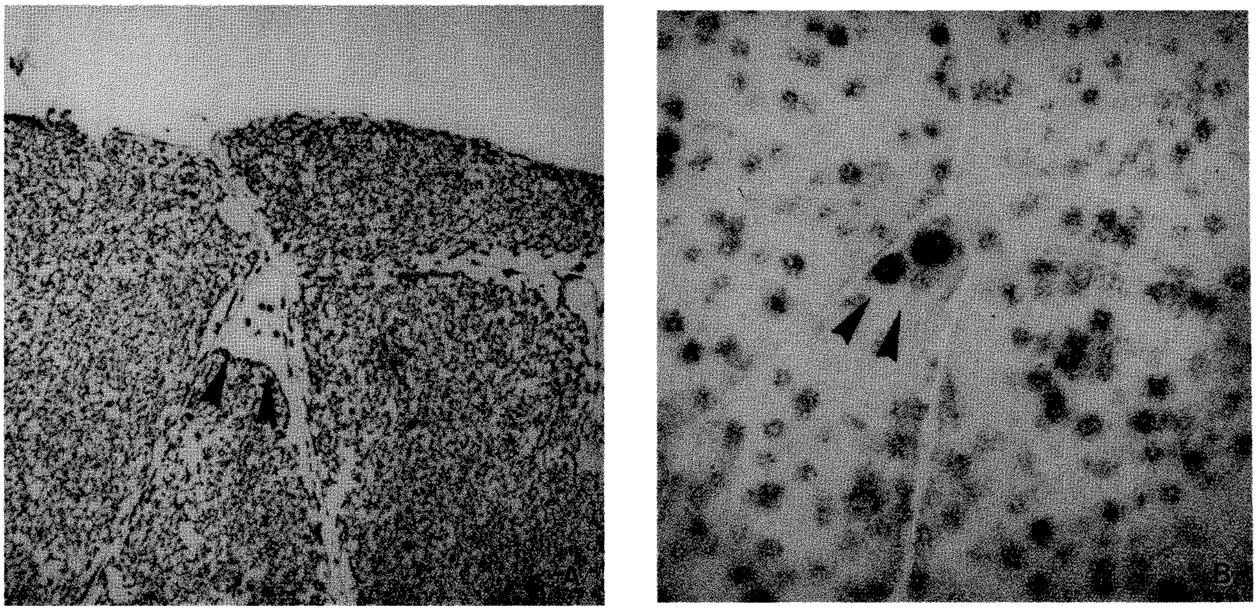

FIGURE 10. Semiserial (1 intervening section omitted) sections of EFA (arrowheads) with a few cells positive for (A), MHC Class-II immunostaining, and (B) strongly positive for nonspecific esterase enzyme-histochemical staining. (×70).

between 8 to 11 weeks old were used. They were kept under conventional laboratory conditions.

### Tissue Sampling and Preparation

Part of the animals were intraperitoneally injected with 15 mg/kg body weight Bromodeoxyuridine, 2 hr before sacrifice. All animals were anesthetized with ether, and bled to death via the abdominal aortia. The thymus was removed, fixed in neutral, phosphate-buffered 4% solution of formaldehyde or snap-frozen in isopentance in liquid nitrogen, and stored at −80°C. Formaldehyde-fixed tissues were embedded in paraffin, sectioned at 5 μm, and stained with H&E. Cryostat sections (5–7 μm) were stained with oil red O and Sudan Black (Pearse, 1968) for lipids.

### Immunohistochemistry

Cryostat sections (5–7 μm) from the thymus were air dried on glass slides, and fixed for 10 min in acetone. Thereafter, they were rinsed in phosphate-buffered saline (PBS, 0.01 M, pH 7.4) and preincubated with 10% normal rabbit serum for 20 min. Serial sections were incubated for 60 min with one of the monoclonal or polyclonal antibodies listed in Table 1. The sections were then rinsed in PBS and layered for 30 min with a peroxidase-conjugated rabbit antimouse Ig (RAMPO, Dakopatts, Denmark), which was diluted in PBS with 4% normal rat serum. The sections were subsequently rinsed in PBS and Tris/HCl (0.05 M, pH 7.6) and finally incubated with the chromogen 3'3'-diaminobenzidine-tetrahydrochloride (Sigma) in a concentration of 0.5 mg/ml in Tris/HCl containing 0.01% $H_2O_2$ for 10 min. The whole procedure was carried out at room temperature. Most sections were slightly counterstained with haematoxylin. Control slides were incubated with the conjugated Ig (RAMPO), PBS, or the chromogen only. No labeling was observed except for a few polymorphonuclear granulocytes when present.

Two-color immunostaining was performed as follows. Sections were incubated with one of the antibodies listed in Table 1, for 60 min. They were rinsed in PBS, and thereafter incubated for 30 min with alkaline phosphatase-conjugated rabbit antimouse immunoglobulins (RAMPh, Dakopatts, Denmark). After rinsing with PBS and Tris/HCl, the sections were incubated with the chromogen naphthol AS-MX phosphate and Fast Blue BB salt for about 15 min. The slides were

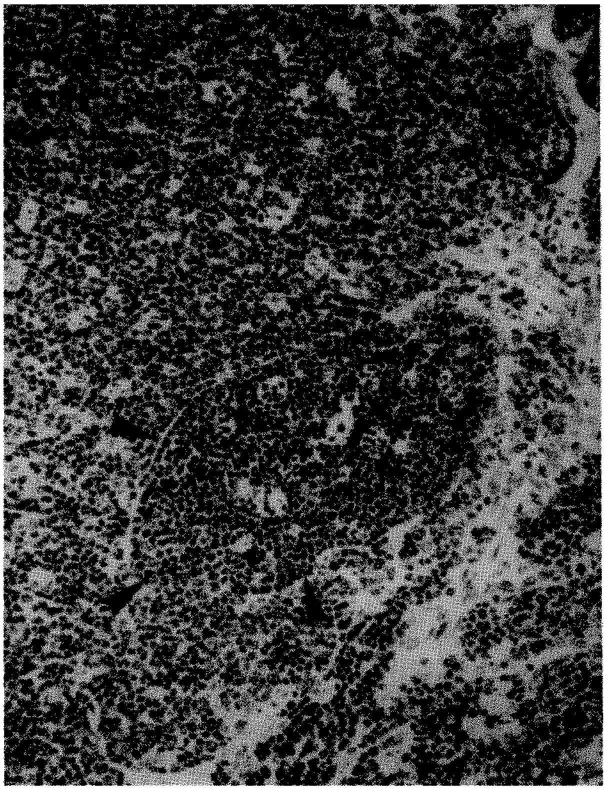

FIGURE 11. Thymus of rat treated with dexamethason (7-days gavage study with 12 mg dexamethason/kg body weight). Tingible body macrophages in EFA (arrowheads) and cortex. H&E staining. (×160).

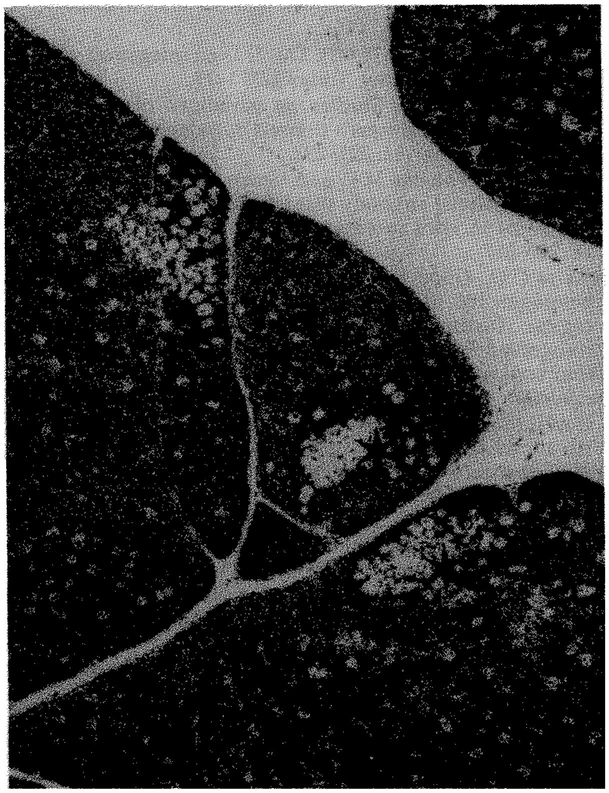

FIGURE 12. Thymus of rat treated with cyclosporin (14-days gavage study with 30 mg cyclosporin/mg body weight). Single, large macrophage like cells and aggregates of these cells in EFA(*). H&E staining. (×70).

rinsed again in PBS, incubated with the second antibody from Table 1, and again rinsed with PBS. Finally, the RAMPh complex was visualized using naphthol AS-BI phosphate and New Fuchsin, for 30 min. The sections were not counterstained.

From one thymus, thirty serial sections (7 μm thick) were made. The slides were stained for MHC Class-II antigen (OX4; see Table 1) to follow the EFA in a block of approximately 0.25 mm.

### Enzyme-Histochemistry

Acid phosphatase activity was demonstrated according to Burstone (Pearse, 1968) with naphthol AS-BI phosphate (Sigma) as the substrate. The incubation time was 30–60 min at 37°C. The substrate for the demonstration of nonspecific esterase was alpha-naphthyl acetate (Sigma; Pearse, 1972). Cryostat sections were used. Incubation time was 10–20 min at room temperature. For both reactions, hexazotized pararosaniline was used as the diazonium salt.

### ACKNOWLEDGMENTS

We acknowledge greatly the gifts of the following monoclonal antibodies: Monoclonal antibodies in the ED series from C.D. Dijkstra, Free University of Amsterdam, The Netherlands; R73 from Th. Hunig, University of Wurzburg, Germany; monoclonal antibodies in the HIS series from J. Kampinga and F.G.M. Kroese, University of Groningen, The Netherlands; and antikeratins from F.C.S. Ramaekers, University Hospital Maastricht, The Netherlands. We thank A. Stenus-van Basten and S. de Vlugt-van den Koedijk for their help in preparation of the manuscript.

*(Received August 11, 1992)*

*(Accepted October 21, 1992)*

### REFERENCES

Boyd R.L., and Hugo P. (1991). Towards an integrated view of thymopoiesis. Immunol. Today **12:** 71–79.

Christensen S. (1952). Studies on variations of the argyrophilic network in the rat's thymus correlated with the age-groups. Acta Anat. **16:** 221–232.

Dallman M.J., Thomas M.J., and Green J.R. (1984). MRC OX-19 a monoclonal antibody that labels rat T lymphocytes and augments in vitro proliferative response. Eur. J. Immunol. **14:** 260–267.

Dijkstra C.D., Döpp E.A., Joling P., and Kraal G. (1985). The heterogeneity of mononuclear phagocytes in lymphoid organs: Distinct macrophage subpopulations in the rat recognized by monoclonal antibodies ED1, ED2, ED3. Immunology **54:** 589–599.

Duijvestijn A.M., Sminia T., Kohler Y.G., Janse E.M., and Hoefsmit E.C.M. (1982). Rat thymus micro-environment: An ultrastructural and functional characterization. *In:* In vivo immunology, Nieuwenhuis P., van den Broek A., and Hanna M.G., Eds. (New York: Plenum), pp. 441–446.

Godfrey D.I., Izon D.J., Tucek C.L., Wilson T.J., and Boyd R.L. (1990). The phenotypic heterogeneity of mouse thymic stromal cells. Immunology **70:** 66–74.

Gratzner H.G. (1982). Monoclonal antibody to 5-bromo- and 5-iododeoxyuridine: A new reagent for detection of DNA replication. Science **218:** 474–475.

Hunig T., Wallny H.J., Hartley J.K., Lawetzky A., and Tiefenthaler G. (1989). A monoclonal antibody to a constant determinant of the rat T cell antigen receptor that induces T cell activation. Differential reactivity with subsets of immature and mature T lymphocytes. J. Exp. Med. **169:** 73–86.

Kampinga J. (1990). Thymocyte differentiation and thymic microenvironment development in the fetal rat thymus: An immunohistological approach. *In:* Thymus update 3: The role of the thymus in tolerance induction, Kendall M.D., and Ritter M.A., Eds. (London: Harwood Academic Publishers), pp. 149–187.

Kampinga J., Kroese F.G.M., Duijvestijn A.M., Murawska M.B., Pol G.H., and Nieuwenhuis P. (1987). The rat thymus microenvironment: Subsets of thymic epithelial cells defined by monoclonal antibodies. Transpl. Proc. **19:** 3171–3174.

Kendall M.D. (1989). The morphology of perivascular spaces in the thymus. Thymus **13:** 157–164.

Kroese F.G.M., Wubbena A.S., Opstelten D., Deenen G.J., Schwander E.H., de Leij L., Vos H., Poppema S., Volberda J., and Nieuwenhuis P. (1987). B lymphocyte differentiation in the rat: Production and characterization of monoclonal antibodies to B lineage associated antigens. Eur. J. Immunol. **17:** 921–928.

Kuper C.F., Beems R.B., Bruijntjes J.P., Schuurman H.-J., and Vos J. (in press). Thymus in adult and old rat. *In:* ILSI monograph on age-associated changes in laboratory animals. The Rat, Mohr U., Capen Ch., and Dungworth D., Eds. (Berlin: Springer Verlag).

McMaster W.R., and Williams A.F. (1979). Identification of Ia glycoproteins in rat thymus and purification from rat spleen. Eur. J. Immunol. **9:** 426–433.

Milicevic N.M. and Milicevic Z. (1989). Histochemistry of the acutely involved thymus in nickel chloride-treated rats. J. Comp. Pathol. **101:** 143–150.

Milicevic N.M., Milicevic Z., Colic M., and Mujovic S. (1987). Ultrastructural study of macrophages in the rat thymus, with special reference to the cortico-medullary zone. J. Anat. 150-89-98.

Moll R., Franke W.W., Schiller D.L., Geiger B., and Krepler R. (1982). The catalog of human cytokeratins: Patterns of expression in normal epithelia, tumors and cultured cells. Cell **32:** 11–24.

Pearse A.G.E. (1968). Histochemistry. Theoretical and applied, vol. I., 3d ed. (New York: Churchill Livingstone).

Pearse A.G.E. (1972). Histochemistry. Theoretical and applied, vol II., 3d ed. (New York: Churchill Livingstone).

Ramaekers F.C.S., Huijsmans A., Schaart G., Moesker O., and Vooijs G. (1987). Tissue distribution of keratin 7 as monitored by a monoclonal antibody. Exp. Cell. Res. **170:** 235–249.

Ramaekers F.C.S., Puts J.J.G., Moesker O., Kant A., Huijsmans A., Haag D., Jap P.H.K., Herman C.J., and Vooijs G.P. (1983). Antibodies to intermediate filament proteins in the immunohistochemical identification of human tumours: An overview. Histochem. J. **15:** 691–713.

Rozing J., Coolen C., Tielen F.J., Weegenaar J., Schuurman H.-J., Greiner D.L., and Rossini A.A. (1989). Defects in the thymic epithelial stroma of diabetes prone BB rats. Thymus **14:** 125–135.

Schuurman H.-J., Van Loveren H., Rozing J., Van Dijk A., Loeber J.G., and Vos J.G. (1990). Cyclosporin and the rat thymus. An immunohistochemical study. Thymus **16:** 235–254.

Shi Y., Sahai B.M., and Green D.R. (1989). Cyclosporin A inhibits activation-induced cell death in T cell hybridomas and thymocytes. Nature **339:** 625–626.

Steinmann G.G. (1986). Changes in the human thymus during aging. *In:* The human thymus, Muller-Hermelink H.K., Ed. (Berlin: Springer Verlag), pp. 43–89.

Van Ewijk W. (1984). Immunohistology of lymphoid and non-lymphoid cells in the thymus in relation to T lymphocyte differentiation. Amer. J. Anat. **170:** 311–330.

Von Gaudecker B. (1986). The development of the human thymus microenvironment. Curr. Topics Pathol. **75:** 1–39.

Williams A.F., Galfre G., and Milstein C. (1977). Analysis of cell surfaces by xenogeneic myeloma-hybrid antibodies. Differentiation antigens of rat lymphocytes. Cell **12:** 633–673.

